# 3D object reconstruction: A comprehensive view-dependent dataset

**DOI:** 10.1016/j.dib.2024.110569

**Published:** 2024-06-02

**Authors:** Rafał Staszak, Dominik Belter

**Affiliations:** Institute of Robotics and Machine Intelligence, Poznan University of Technology, Pl. Marii Sklodowskiej-Curie 5, 60-965 Poznan, PL, Poland

**Keywords:** Robotics, RGB-D camera, Depth images, Single-view scene reconstruction, Scene segmentation, Grasping objects

## Abstract

The dataset contains RGB, depth, segmentation images of the scenes and information about the camera poses that can be used to create a full 3D model of the scene and develop methods that reconstruct objects from a single RGB-D camera view. Data were collected in the custom simulator that loads random graspable objects and random tables from the ShapeNet dataset. The graspable object is placed above the table in a random position. Then, the scene is simulated using the PhysX engine to make sure that the scene is physically plausible. The simulator captures images of the scene from a random pose and then takes the second image from the camera pose that is on the opposite side of the scene. The second subset was created using Kinect Azure and a set of real objects located on the ArUco board that was used to estimate the camera pose.

Specifications Table

**Table:** 

Subject	Computer Science
Specific subject area	Computer Science Applications
Data format	*Raw RGB and depth images taken from two random but correlated camera poses for each random scene containing an object from the ShapeNet dataset.*
Type of data	8-bit RGB images, 16-bit depth images, 8-bit segmentation images, csv files containing information about the scene and camera poses for 3D reconstruction, yaml files containing information about the camera, objects on the scene, and IMU data.
Data collection	Data were collected in the custom simulator that loads random graspable objects from the ShapeNet dataset and random table. A graspable object is placed above the table in a random position. Then, the scene is simulated using the PhysX engine to make sure that the scene is physically plausible. The simulator captures images of the scene from a random pose and then takes the second image from the camera pose that is on the opposite side of the scene. The dataset also contains RGB-D images from the Kinect Azure camera collected with real objects from the YCB dataset.
Data source location	Poznan University of Technology, *ul. Piotrowo 3A, PL 60–965 Poznan, Poland,* *GPS: 52.402522353291126, 16.9536038416983*
Data accessibility	*Repository name: A Comprehensive View-Dependent Dataset for Objects Reconstruction - Synthetic Set Part A* *Data identification number:*10.17632/z88tpm3926.2 10.17632/hy9wnbhr9w.3 10.17632/jd8w5r3ncw.2 *Direct URL to data:* https://data.mendeley.com/datasets/z88tpm3926/1 https://data.mendeley.com/datasets/hy9wnbhr9w/2 https://data.mendeley.com/datasets/jd8w5r3ncw/1
Related research article	*R. Staszak, B. Kulecki, W. Sempruch, D. Belter, What's on the Other Side? A Single-View 3D Scene Reconstruction, 2022 17th International Conference on Control, Automation, Robotics and Vision (ICARCV), 173–180, 2022.*

## 1 Value of the Data

• This dataset was collected in a controlled environment and it provides ground truth RGB and depth images of the scenes.
• The dataset also contains real RGB-D images from the Kinect Azure captured with the camera pose while the sensor was moving around the scene.
• The dataset also contains ground truth synthetic data related to object segmentation and object position.
• The dataset can be used to reconstruct occluded parts of the objects and the scene.
• The dataset can also be used for scene segmentation, object detection, and pose estimation using RGB-D images.
• The dataset might be used in practical tasks combining different aspects of object reconstruction and detection, domain adaptation, robotic manipulation, and synthetic-to-real transfer learning.

## 2 Background

**Fig. 1:**
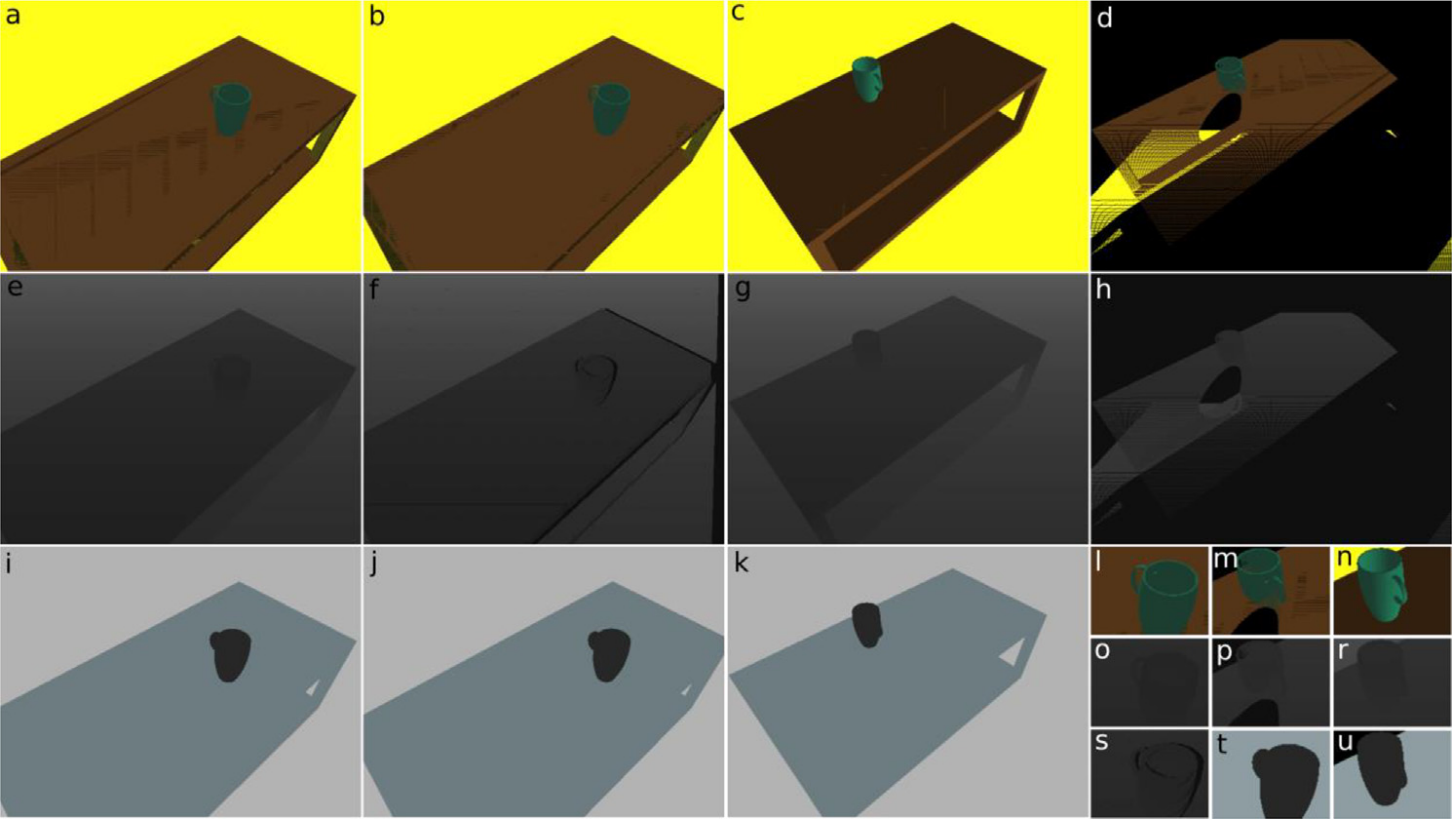
Example images from the dataset for a random scene. The resolution of the images is 640 × 480 (a–k) and 129 × 96 (l–u). See the text for a detailed explanation.

The motivation behind creating the dataset stems from the challenges that robots face in perceiving and reconstructing objects from a single viewpoint. This limitation often leads to incomplete shape information about the objects, negatively impacting the effectiveness of grasping methods. The incomplete or partial object models limit the robotʼs ability to interact with objects in any given environment. To address this issue, we developed a dataset that contains RGB-D images of the scene from the input pose and the pose on the opposite side of the scene. Both RGB-D pairs of images and camera poses can be used to create a 3D model of the scene (point cloud). The motivation for creating the dataset lies in improving the perceptual capabilities of robots, specifically in the context of single-view object reconstruction, with the aim of enhancing their ability to interact with and manipulate objects in various environments. By creating a dataset specifically tailored to the challenges posed by single-view object reconstruction, the goal is to enable robots equipped with RGB-D cameras to overcome the limitations associated with incomplete shape information [1] (Fig. 1).

## 3 Data Description

**Fig. 2:**
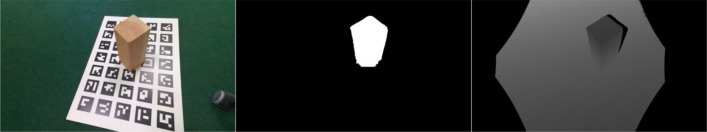
Example RGB (a), object mask (b), and depth images (c) from the dataset of real objects.

The first subset of the dataset represents computer-generated scenes, where a single object, which stands on a table in a random position, is captured from various viewpoints. The dataset contains synthetic RGB-D images and corresponding 3D camera poses [2,3]. The pairs of RGB-D images are generated for the random initial pose of the camera and the pose on the other side of the scene. The images stored in the dataset are presented in Fig. 2, where we show the RGB image for the random pose of the camera (a), the RGB image aligned with the depth camera pose (b), the RGB image from the pose of the camera located on the opposite side of the scene (c), RGB image obtained by projecting the point cloud from the random camera pose on the pose of the camera located on the other side of the scene (d), corresponding depth images (e–h), and corresponding segmentation images (i-k). We also store 128×96 px patches cropped from the original scene that contain single objects (Fig. 2–u).

The images stored in the dataset are grouped according to the category name. Inside each folder, the images are identified according to the content (RGB, depth, segment, RGBprojected, depthProjected). The number of the image represents the identifier of the camera pose. The camPoses.csv file contains information about camera poses used to collect the data set. Each image name contains the identifier of the camera pose that is represented as a single row in the CSV file. In each row we store the identifier of the camera pose, the identifier of the initial random camera pose, and row-wise elements of the homogenous transformation matrix related to the camera pose. Moreover, for each scene, the dataset provides an objects.dat file that contains the names of the objects on the images, instances identifiers from the ShapeNet dataset, a region identifier on the segmentation images, image coordinates, and row-wise elements of the homogenous transformation matrix related to the object's pose.

**Fig. 3:**
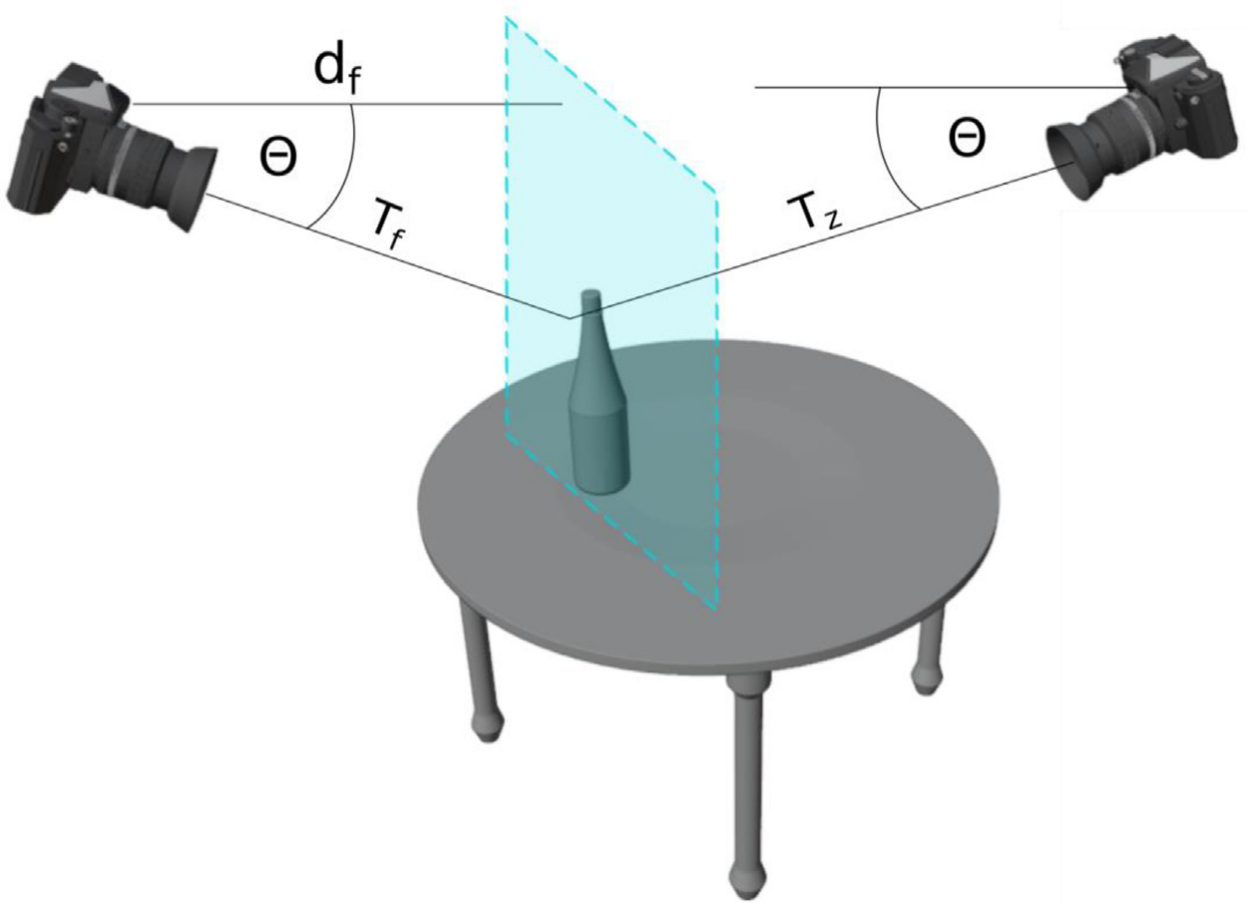
Illustration of the virtual camera pose (right) that is on the opposite side of the scene to the input view (left). The translation T_z_ is constant and the virtual surface of the mirror does not have to be in the center or aligned to the surface of the object.

The second subset [4] utilizes a set of eight YCB objects [5] augmented with two objects - a bottle and a wooden box. Single objects are put on a 60 × 40 cm board with Aruco markers and Multiple shots from different viewpoints are taken around them. To capture data, the Kinect Azure DK sensor was employed due to its superior depth data quality and density when compared to other cameras [6]. Example images are presented in Fig. 3. The dataset contains the following files:

­ RGB images (“rgb_” prefix) - RGB images of the scene, resolution: 1920×1080,
­ camera poses in the board frame (“board_” prefix) – homogeneous matrix in row-major order,
­ correction of the camera poses (“correction_” prefix) – homogeneous matrix in row-major order,
­ 16-bit depth images of the scene (“depth_” prefix) – resolution: 1920×1080,
­ 16-bit undistorted depth images of the scene (“depth_undist_” prefix) – resolution: 1920×1080,
­ Inertial Measurement Unit (IMU) data from the camera: orientation, angular velocity, linear acceleration in yaml format (“imu_” prefix),
­ estimated position of the Aruco markers (“markers_” prefix),
­ object mask images (“mask” prefix) – resolution 1920×1080,

The corrected camera pose **C** is computed using the formula:

$$C\;={\left(T_{B}\;T_{C}^{-1}\right)}^{-1},$$

where **T**_B_ is the estimated camera pose with respect to the Aruco board and **T**_C_ is the correction of the camera pose.

## 4 Experimental Design, Materials and Methods

In Fig. 3 we illustrate the data acquisition process. The initial and random camera pose is located randomly around the table. The inclination of the camera is randomly drawn from the range of 0.7–0.9 rad, the yaw angle is drawn from the range of -Π to Π. The position of the camera is computed from these spherical coordinates assuming that the radiance is equal to 2.5 m. The "mirror" pose of the camera shown on the right in Fig. 3 is computed relatively to the plane associated with the front surface of the object, represented by the blue plane in Fig. 3. We keep a relatively constant distance between the virtual camera pose by employing a constant translation equal to 1.3 m, denoted as **T**_z_. The mirror camera pose, depicted on the right in Fig. 3, is derived through the application of defined homogeneous transformations to the input camera pose:

$$T=\;T_{f}\;T_{\Theta }^{-1}\;T_{yaw=\Pi }\;T_{\Theta }\;T_{z}^{-1},$$

where **T**_f_ is the transformation along the z-axis of the camera that depends on the distance between the current camera pose and the front surface of the object, **T**_Θ_ is the transformation that compensates current inclination Θ (pitch angle) of the camera, and **T**_yaw=Π_ is the transformation that moves the camera to the other side of the scene that is 1.3 m distant from the front surface of the object (T_z_ transformation). Finally, the virtual camera pose depends only on the tilt of the camera and the distance between the camera and the front surface of the object d_z_. The other parameters are fixed. Even though we assume that the virtual camera pose is 1.3 m distant from the front surface of the object, it may vary due to imprecise front surface estimation.

On the table, we place one random instance of an object selected from a random set of categories (bottle, camera, can, jar, laptop, mug, bowl, box from the ShapeNet [7]). The horizontal position of the object is randomly selected from a uniform distribution in the range of −0.25 to 0.25 m. Upon selecting a table randomly for a given object, the data generation procedure starts for the particular scene. Moreover, the scenes are constrained by a surface that represents a floor. The geometrical size of objects is scaled down by a factor of 0.25, while the tables have been scaled up by a factor of 1.5 to mimic natural proportions. The scene dimensions lie between3.0 m and 4.0 m depending on the assumed viewpoint. Depth images are generated using an OpenGL graphics engine and the pinhole camera model. The parameters of the camera are based on the perfect camera model (f_x_=f_y_=525, C_x_=320, C_y_=240). Moreover, we generate reference depth image and depth image that is created assuming that RGB and depth cameras are not aligned like in real RGB-D cameras. The translation between these cameras is equal to t_x_=−0.051, t_y_=0.001, and t_z_=−0.001 and they are obtained from calibrating our Kinect Azure camera.

The subset containing images from the Kinect Azure (fx=912.37, fy=912.25, Cx=961.44, Cy=548.29) was collected by moving the camera above a set of real objects located on the ArUco 60 × 40 cm board that was used to estimate the camera pose. To estimate the camera poses for the objects from the subset of real objects, we used the ArUco board, which consists of a grid of 7 × 5 unique ArUco markers. We placed the object on the ArUco board and utilized the OpenCV library methods to estimate the camera poses [8] located around the object and the marker.

The obtained views from multiple poses can be used to construct a 3D point cloud model of the observed object, subsequently employing it to generate RGB and depth images based on the provided camera pose. This process involves merging point clouds generated from various viewpoints surrounding the observed objects. The precision of the acquired 3D model of the object is heavily dependent on the accuracy of the camera pose estimation. Even a slight deviation can lead to sets of point clouds from different viewpoints that do not perfectly align, especially when relying solely on RGB data. To address this, we utilize the Broyden–Fletcher–Goldfarb–Shanno (BFGS) algorithm [9] to refine the board poses based on depth measurements. The corners of detected ArUco markers are assigned to corresponding points in space based on the depth data. Hence, it is possible to refine the initial board pose by minimizing the distance between the known board layout to the corresponding points in space. Despite the initial ArUco-based localization and subsequent BFGS-based correction, the resulting point clouds may not align flawlessly. Consequently, a manual alignment of the point clouds in 3D space is performed to achieve the ground-truth models. The generation of views from arbitrary viewpoints is possible by merging selected samples, which are assigned to a particular object. The selection involves reducing the number of dataset views used for the synthesis of a partial point cloud by comparing the cosine distance between the z-vectors of the newly defined camera viewpoint and the dataset camera viewpoints. Then, the data samples can be sorted in descending order of the obtained distances and the first few occurrences are used to obtain RGB and depth images for the given camera pose.

## Limitations

The dataset focuses on depth data. The RGB images in the synthetic dataset are generated using very simple renderers and are used for visualization purposes and should not be used for training neural networks.

## Ethics Statement

We confirm that the current work does not involve human subjects, animal experiments, or any data collected from social media platforms.

## CRediT Author Statement

**Rafał Staszak:** Software, Validation, Real-world Experiments, Reviewing and Editing **Dominik Belter:** Simulation software, Original draft preparation, Supervision

## Data Availability

A Comprehensive View-Dependent Dataset for Objects Reconstruction - Kinect Azure Set (Original data) (Mendeley Data). A Comprehensive View-Dependent Dataset for Objects Reconstruction - Synthetic Set Part A (Original data) (Mendeley Data). A Comprehensive View-Dependent Dataset for Objects Reconstruction - Synthetic Set Part B (Original data) (Mendeley Data).
